# Supplementary material for: Effect of Treadmill Exercise and Trans-Cinnamaldehyde against d-Galactose- and Aluminum Chloride-Induced Cognitive Dysfunction in Mice
Source: Brain Sci. 2020 Oct 29;10(11):793. doi: 10.3390/brainsci10110793 (PMC7693345; doi:10.3390/brainsci10110793)

Fig S1. Original images for blot of Nrf2, NQO1, HO-1 and SOD-1

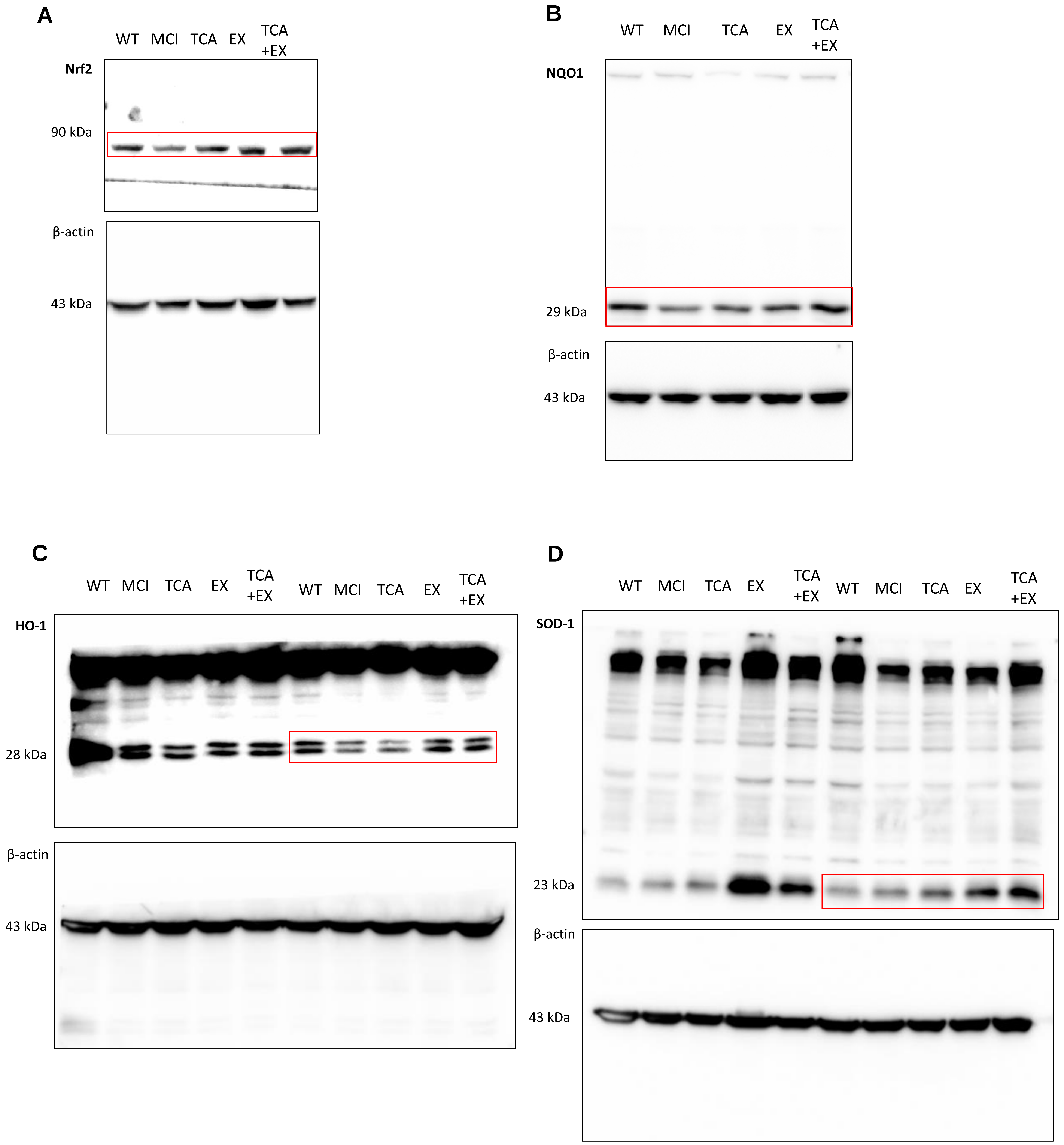

Fig S2. Original images for blot of p-AMPK and PGC1-α

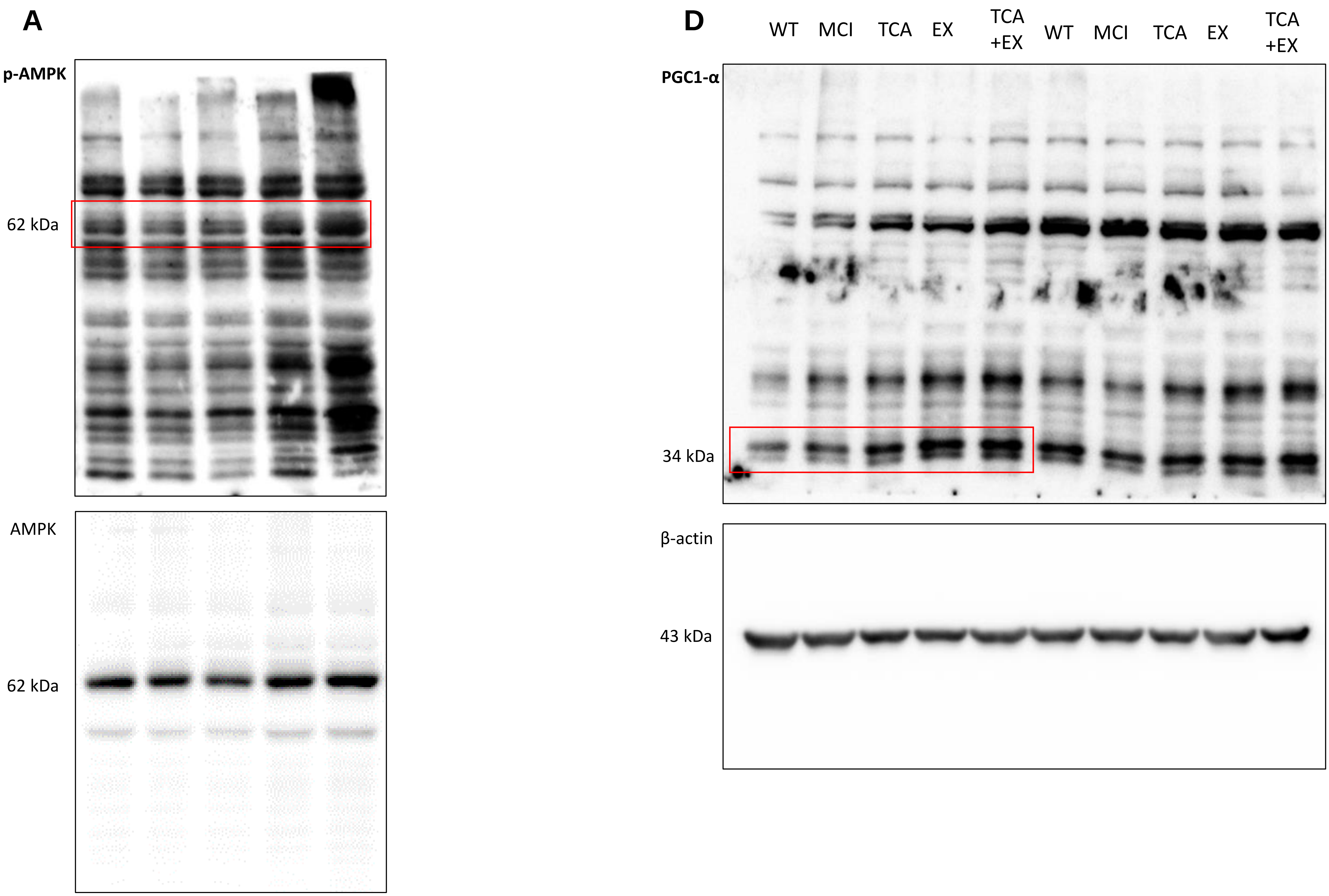

Supplement: Supplementary file 1 [file brainsci-10-00793-s001.pdf]
